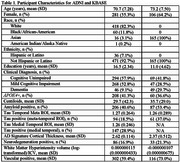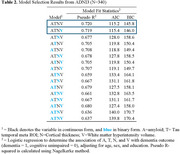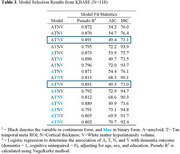# Cross‐National Study to Operationalize Amyloid, Tau, Neurodegeneration, and Vascular Contributions to Dementia

**DOI:** 10.1002/alz70856_106635

**Published:** 2026-01-11

**Authors:** Jeremy A. Tanner, Diefei Chen, Min Soo Byun, Evgeny J. Chumin, Ileana De Anda‐Duran, Kacie D Deters, Martine Elbejjani, Evan Fletcher, A. Zarina Kraal, Dong Young Lee, Silvia Mejia‐Arango, Stefanie Pina‐Escudero, Kwangsik Nho, Talia L. Robinson, C. Elizabeth Shaaban, Adam M. Staffaroni, Andrew J. Saykin, Paul K Crane, Dahyun Yi, Shannon L Risacher, the Alzheimer's Disease Neuroimaging Initiative; the KBASE Research Group

**Affiliations:** ^1^ University of Texas Health San Antonio, San Antonio, TX, USA; ^2^ Department of Epidemiology, Bloomberg School of Public Health, Johns Hopkins University, Baltimore, MD, USA; ^3^ Department of Neuropsychiatry, Seoul National University Hospital, Seoul, Korea, Republic of (South); ^4^ Indiana Alzheimer's Disease Research Center, Indiana University School of Medicine, Indianapolis, IN, USA; ^5^ Center for Neuroimaging, Department of Radiology and Imaging Sciences, Indiana University School of Medicine, Indianapolis, IN, USA; ^6^ Celia Scott Weatherhead Tulane University School of Public Health and Tropical Medicine, New Orleans, LA, USA; ^7^ University of California Los Angeles, Los Angeles, CA, USA; ^8^ American University of Beirut, Beirut, Beirut, Lebanon; ^9^ Department of Neurology, University of California, Davis, Davis, CA, USA; ^10^ Taub Institute for Research on Alzheimer's Disease and the Aging Brain, New York, NY, USA; ^11^ Columbia University Irving Medical Center, New York, NY, USA; ^12^ Seoul National University Medical Research Center, Seoul, Korea, Republic of (South); ^13^ Interdisciplinary program of cognitive science, Seoul National University College of Humanities, Seoul, Korea, Republic of (South); ^14^ Department of Psychiatry, Seoul National University College of Medicine, Seoul, Korea, Republic of (South); ^15^ RGV Alzheimer's Resource Center (AD‐RCMAR), Harlingen, TX, USA; ^16^ The University of Texas Rio Grande Valley School of Medicine, Harlingen, TX, USA; ^17^ Global Brain Health Institute (GBHI), University of California San Francisco (UCSF); & Trinity College Dublin, San Francisco, CA, USA; ^18^ Department of Radiology and Imaging Sciences, Indiana Alzheimer's Disease Research Center, Center for Neuroimaging, Indiana University School of Medicine, Indianapolis, IN, USA; ^19^ Brigham and Women's Hospital, Boston, MA, USA; ^20^ Massachusetts General Hospital, Boston, MA, USA; ^21^ University of Pittsburgh Alzheimer's Disease Research Center (ADRC), Pittsburgh, PA, USA; ^22^ Memory and Aging Center, UCSF Weill Institute for Neurosciences, University of California, San Francisco, San Francisco, CA, USA; ^23^ Department of General Internal Medicine, University of Washington School of Medicine, Seattle, WA, USA; ^24^ Indiana Alzheimer's Disease Research Center, Indiana University School of Medicine, Indianapolis, IN, USA

## Abstract

**Background:**

Recent diagnosis and staging criteria have been proposed for AD based on amyloid(“A”), tau(“T”), and neurodegeneration(“N”), with consideration of comorbid vascular(“V”) pathology. However, challenges remain in their application and interpretation for research and clinical use. Additionally, current criteria have been largely informed by data derived from samples of highly educated, non‐Hispanic White cohorts in the US. We compared methods to operationalize multimodal ATNV measures across two cohorts to meaningfully inform future research and clinical practice.

**Method:**

Participants with amyloid PET, tau PET, and brain MRI were included from two prospective cohort studies with similar study designs: ADNI3 in the US and KBASE in Korea. ATNV measures were operationalized as continuous (A=centiloid; T=meta‐temporal ROI; *N* = AD signature region cortical thickness; V=white matter hyperintensity volume) and as binary (+/‐) variables by replicating approaches applied in other cohorts. Clinical diagnoses of cognitively unimpaired (CU), mild cognitive impairment (MCI), and dementia were determined by experts at multidisciplinary consensus conferences. Parallel cross‐sectional analyses were performed within each cohort. Multivariate logistic regressions with ATNV modeled as continuous or binary predictors (16 possible combinations) adjusting for age, sex, and education were performed to compare model fit and predictive power for distinguishing participants with dementia versus CU.

**Result:**

A total of 508 participants in ADNI (mean age=71±7, female=55%, education(yrs)=16.5±2.3) and 165 in KBASE (*n* = 165; age=73±8, female=64%, education(yrs)=11.0±4.6) were included (Table 1). In ADNI, the continuous ATNV and continuous ATN+binary V explained the most variance in dementia diagnosis (highest pseudo‐R^2^, lowest AIC/BIC; Table 2). In KBASE, models had higher overall predictive power for distinguishing dementia. The best‐performing models included continuous A and T, binary N, and continuous V, or binary A, continuous T, binary N, and continuous V (Table 3).

**Conclusion:**

Models including ATNV were highly predictive of dementia in both cohorts. Models with predominantly continuous variables explained more variability in dementia diagnosis, and are optimal for research use due to their simplicity. In Korea, binary N and binary A increased predictive power. Ongoing analyses will assess the relative contribution of ATNV to cognitive performance and diagnosis in each cohort to better inform appropriate research and clinical use in cross‐national cohorts.